# Dapagliflozin Modulates the Fecal Microbiota in a Type 2 Diabetic Rat Model

**DOI:** 10.3389/fendo.2020.00635

**Published:** 2020-11-17

**Authors:** Mei Yang, Fang-Hong Shi, Wen Liu, Min-Chun Zhang, Ri-Lu Feng, Cheng Qian, Wei Liu, Jing Ma

**Affiliations:** ^1^Department of Endocrinology and Metabolism, Renji Hospital, School of Medicine, Shanghai Jiao Tong University, Shanghai, China; ^2^Department of Pharmacy, Renji Hospital, School of Medicine, Shanghai Jiao Tong University, Shanghai, China

**Keywords:** dapagliflozin, fecal microbiota, type 2 diabetes, metfomiin, high-fat diet

## Abstract

**Background:** The gut microbiota is recognized as a major modulator of metabolic disorders such as type 2 diabetes. Dapagliflozin, sodium glucose cotransporter 2 inhibitors (SGLT2i), enhances renal glucose excretion, and lowers blood glucose levels. The study aimed to determine the effects of dapagliflozin on fecal microbiota in a type 2 diabetic rat model.

**Methods:** Four-week-old male Sprague Dawley rats (*n* = 24) were fed a high-fat diet (HFD) for 8 weeks and then given a single dose of STZ injection (30 mg/kg, i.p). They were randomly divided into three groups (*n* = 8). Each group received intragastric infusion of normal saline (2 ml, 0.9%) or metformin (215.15 mg/kg/day) or dapagliflozin (1 mg/kg/day) for 4 weeks. Blood glucose levels and plasma insulin levels were detected during intragastric glucose tolerance. Fecal samples were collected to access microbiome by 16S ribosomal RNA gene sequencing.

**Results:** Dapagliflozin significantly decreased fasting and postprandial blood glucose levels as metformin in type 2 diabetic rats (*P* < 0.001). Enterotype was composed of *Ruminococcaceae* after treatment of dapagliflozin, whereas *Ruminococcaceae* and *Muribaculaceae* were the main enterotypes following metformin treatment. Dapagliflozin did not increase the abundance of beneficial bacteria including *Lactobacillaceae* and *Bifidobacteriaceae*. However, these were increased in the metformin group. It is surprising to find that *Proteobacteria* (especially *Desulfovibrionaceae*) were enriched in the dapagliflozin group.

**Conclusion:** Dapagliflozin and metformin exerted complementary effects on the main beneficial bacteria. A combination of these two drugs might be beneficial to improve the structure of fecal microbiota in the treatment of type 2 diabetes.

## Introduction

Type 2 diabetes (T2D) affects nearly 373.5 million individuals worldwide. An increasing number of studies have connected the gut microbiome with metabolic diseases such as diabetes and obesity (1). It has been reported that there was a decreased amount of beneficial bacteria in patients with type 2 diabetes (2). Fecal microbiota transplantation holds potential as a promising option in the treatment of diabetes (3).

The composition and richness of intestinal flora are unique and highly variable. It can be modulated by diet, host health, age, ethnicity, genetics, and antidiabetic medication (4). Metformin is a first-line medication in the treatment of type 2 diabetes, as it increases the abundance of beneficial bacteria (4, 5), and is associated with higher levels of short-chain fatty acids (SCFAs: acetate, propionate, and butyrate) (6). SCFAs conferred beneficial metabolic effects, including improving body weight and glycemic management (7). Treatment of metformin could restore sodium glucose cotransporter 1 (SGLT1) expression and glucose sensing to alter the upper small intestinal microbiota (8).

Sodium glucose cotransporter 2 (SGLT2) inhibitors are used to achieve the glucose-lowering effect by increasing urinary glucose excretion (9). SGLT2 is mainly expressed in the proximal tubule of the kidney, while SGLT1 is highly expressed in the gut (9). SGLT2 inhibitors could act on SGLT1 to reduce intestinal glucose uptake (10). SGLT2 inhibitors decreased the glucose excursion and increased glucagon-like peptide-1 (GLP-1) and peptide YY secretion following carbohydrates (CHO) ingestion (11). The latter may augment glucose exposure of the colonic microbiota and formation of metabolites (11). It has been shown that canagliflozin altered the intestinal microbiota composition in mice with chronic kidney disease (12). The effects of dapagliflozin on fecal microbiota remain controversial. A recent study demonstrated that dapagliflozin altered the fecal microbiota in diabetic mice (13). However, another human study reported that dapagliflozin treatment had no effects on fecal microbiota. It should be noted that it was a combinative effect of metformin and dapagliflozin. Furthermore, there was no placebo in the study (14). Therefore, we aimed to explore the effects of dapagliflozin on fecal microbiota in a type 2 diabetic rat model.

## Methods

### Animals and Ethics Statement

Four-week-old male Sprague Dawley (SD) rats weighing 200–250 g were purchased from Shanghai SLAC Laboratory Animal (Shanghai, China, SCXK2017-0005, SYXK2017-0008). All the SD rats were housed in a controlled environment with a temperature of (20–25°C) and humidity of (40–60%) and allowed free access to water and food. Animal handling and experimental procedures were consistent with the regulations of the Shanghai Government and the Principles of Laboratory Animal Care, published by the US National Institutes of Health (publication no.85-23, revised 1996).

The detailed animal ethics including the animal tests and its handing procedures were approved by the institutional animal care and use committee of SLAC.

### Experimental Protocol

Rats were fed with a high-fat diet for 8 weeks, followed by a single dose of injection of STZ (30 mg/kg, ip). The high-fat diet (HFD) contained 20% protein, 20% carbohydrate, and 60% fat. The blood glucose was detected about 3–7 days after STZ administration. Rats were considered the model of type 2 diabetes mellitus when they met the following criteria (the highest random blood glucose concentrations were higher than 16.7 mmol/L, and the 2 h postprandial glucose concentrations were higher than 11.1 mmol/L). They were randomly divided into three groups (*n* = 8): control, metformin, and dapagliflozin. All the rats received daily gastric gavage with 2 ml saline (0.9%), or metformin (215.15 mg/kg/day, Sino-American Shanghai Squibb Pharmaceuticals, H20023370) or dapagliflozin (1 mg/kg/day, AstraZeneca, H20170117) for 4 weeks. The formula used for the dosage of dapagliflozin and metformin was: drat=dhuman^*^0.71/0.11 (15).

### Intragastric Glucose Tolerance Test (IGGTT)

After 4 weeks of dosage, overnight-fasted rats were given 50% glucose at a dose of 2 g/kg by intragastric administration. Blood samples were collected from the tip of the rats' tails to test glucose levels at 0, 30, 60, 120, and 180 min by using a glucose meter (Accu-Chek, Roche, Swiss). The samples were centrifuged for 10 min at 3,000 rpm (4°C) and plasma was used for insulin measurement.

### DNA Extraction and 16S rRNA Gene Sequencing

We performed 16S rRNA sequencing including fecal sample collection, DNA extraction, and PCR amplification; Illumina MiSeq sequencing and processing of data (16). Bacterial genomic DNA was performed by using the EZNA soil DNA Kit (Omega Bio-tek, Norcross, GA, USA). The 16S rRNA V3-V4 region was amplified via PCR and then sequenced by the Illumina MiSeq platform (Illumina, San Diego, USA).

### Bioinformatic Analysis and 16S rRNA Gene Sequencing

We used bioinformatic analysis and 16S rRNA sequencing mainly including the clustering of microbial community types and metabolic function prediction (16). We analyzed microbial community types by investigating operational taxonomic units (OTU) level microbial alpha (Sobs, Shannon, Ace, and Chao1) and beta diversity, principal coordinates analysis (PCoA), and analysis metabolic function prediction by using Phylogenetic Investigation of Communities by Reconstruction of Unobserved States (PICRUSt).

### Statistical Analysis

SPSS Statistics V 23.0 software (IBM, Chicago, IL, USA) and the online platform Majorbio I-Sanger Cloud Platform were used to analyze the results. Blood glucose was calculated by repeated measures analysis of variance (ANOVA) and expressed as the mean ± SEM. The area under the curve (AUC) was calculated by the trapezoidal method (17). HOMA-IR was calculated by the following formula: HOMA-IR= Fasting blood glucose (FBG, mmol/L) × Fasting insulin (FINS, ng/ml)/22.5.

Indexes of alpha diversity (Sobs, Shannon, Ace, and Chao1) were analyzed by the Kruskal-Wallis test. Beta diversity was calculated by the pMANOVA analysis based on Bray Curtis distances. Kruskal-Wallis sum rank test was used to detect the differences of the relative abundance of bacteria between the groups, including the LEfSe algorithm. Only functional categories reaching a log LDA significant threshold value of >2.0 were shown. Spearman's correlations were calculated between the abundance of bacterial families and blood glucose levels during IGGTT. *P* < 0.05 was considered *s*tatistically significant.

## Results

### Blood Glucose Levels During IGGTT

Fasting blood glucose levels were significantly decreased after metformin and dapagliflozin treatments compared with the control (*P* < 0.001, respectively), with no statistical differences between metformin and dapagliflozin treatments. Postload blood glucose levels in the metformin and dapagliflozin group were markedly decreased at 30, 60, 120, and 180 min (Supplementary Figures 1A,B) compared to the control. There were no significant differences in postload blood glucose levels between metformin and dapagliflozin. Dapagliflozin decreased the levels of HOMA-IR when compared with metformin and the control (Supplementary Figure 1C). There was no difference between metformin treatment and the placebo.

### Fecal Microbiota Diversity

**Figure 1:**
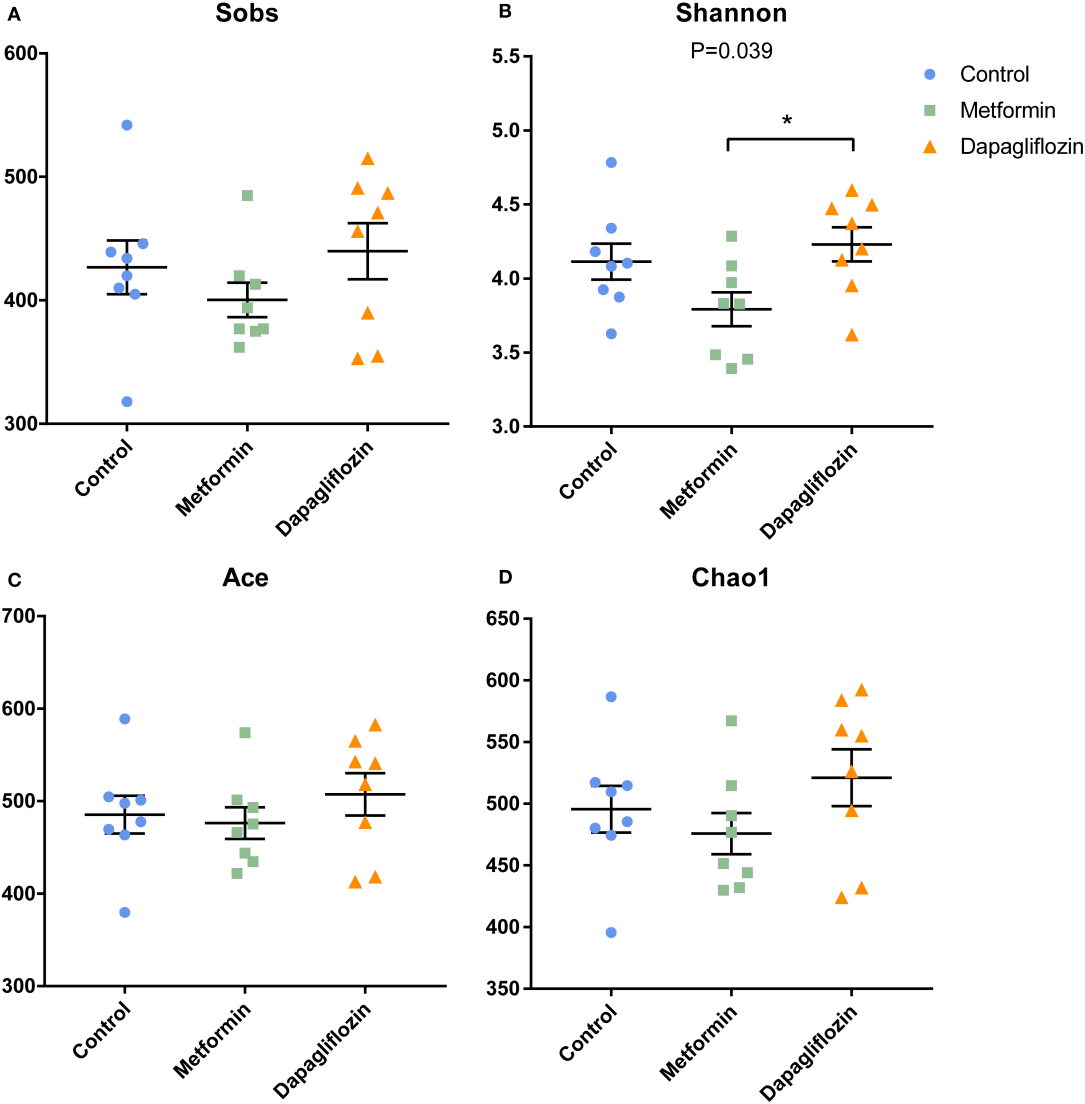
Indexes of alpha diversity in three groups. **(A)** Sobs **(B)** Shannon **(C)** Ace **(D)** Chao1. Data were expressed as mean ± SEM. *P < 0.05.

**Figure 2:**
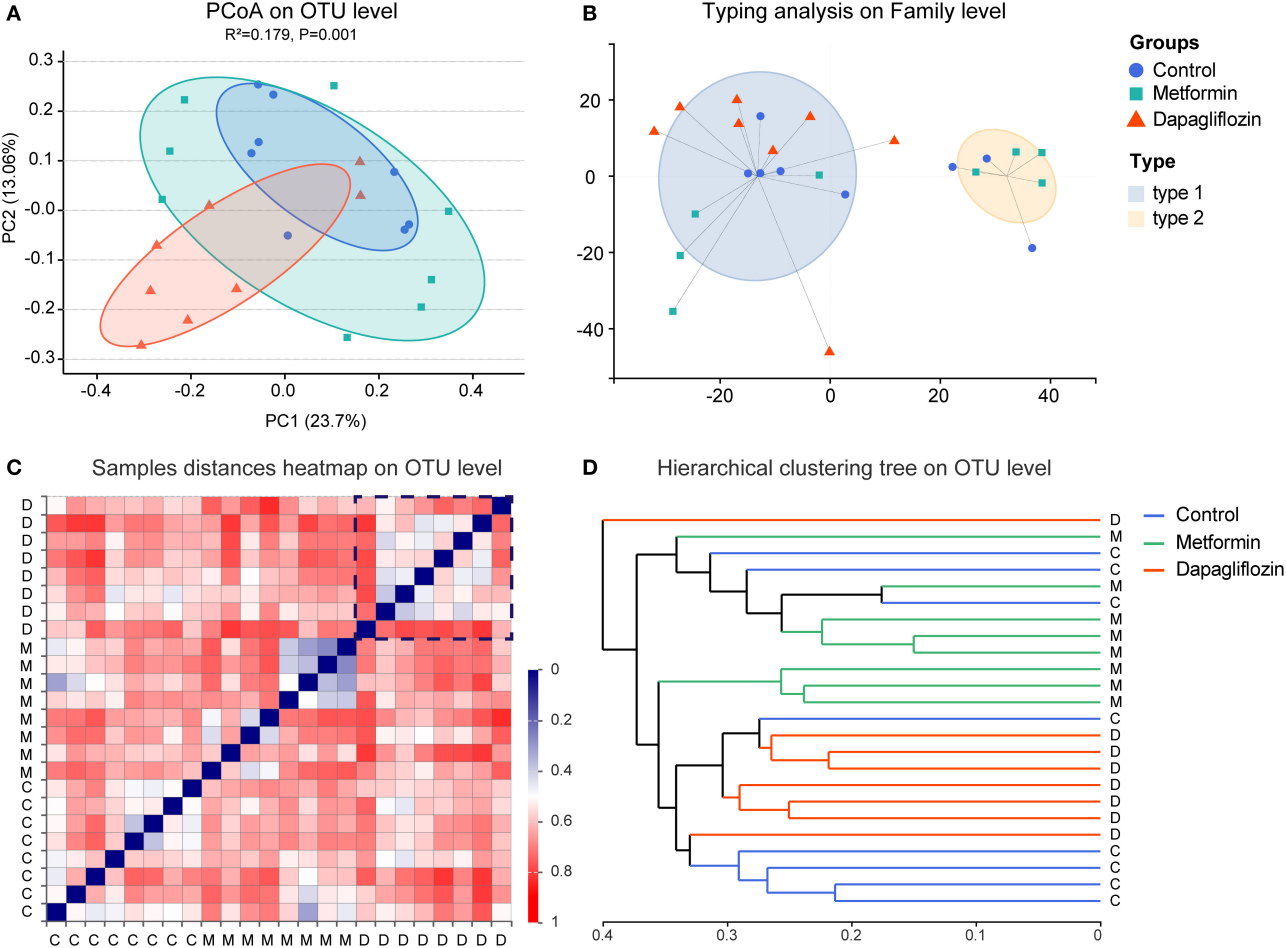
Beta diversity and enterotypes analysis in three groups. **(A)** Principal coordinates analysis (PCoA). The two principal coordinates (PC1-PC2) explain 23.7 and 13.06%, respectively, and the analysis of permutational multivariate analysis of variance (pANOVA) was *p* = 0.001. **(B)** Clustering of all 24 samples into two enterotypes on the family level. Type1 was presented by *Ruminococcaceae* and type 2 was *Muribaculaceae*. **(C)** Samples distance heatmap on operational taxonomic units (OTU) level. Clustering of samples based on Bray-Curtis distances. Each row or column represents one sample. **(D)** Hierarchical clustering tree on OTU level. One straight line represents on sample. C, control group; M, metformin group; D, dapagliflozin group.

The shannon index of dapagliflozin treatment was higher than that of metformin (*P* = 0.039) (Figure 1B). No statistical difference was found in the indexes indicating richness, including Sobs, Ace, and Chao1 (Figures 1A,C,D). The pMANOVA analysis, which was based on Bray Curtis distances, revealed significant alterations of the microbiota communities between metformin, dapagliflozin, and control rats (*P* = *0.001*) (Figure 2A).

The composition of fecal microbiota was distinguished into two enterotypes (Figure 2B). *Ruminococcaceae* was the dominant enterotype in the dapagliflozin group, while the metformin group displayed enterotypes mainly composed by *Ruminococcaceae* and *Muribaculaceae*. The clustering analysis showed that there was more consistency in the dapagliflozin group (Figures 2C,D).

### The Composition of Fecal Microbiota

**Figure 3:**
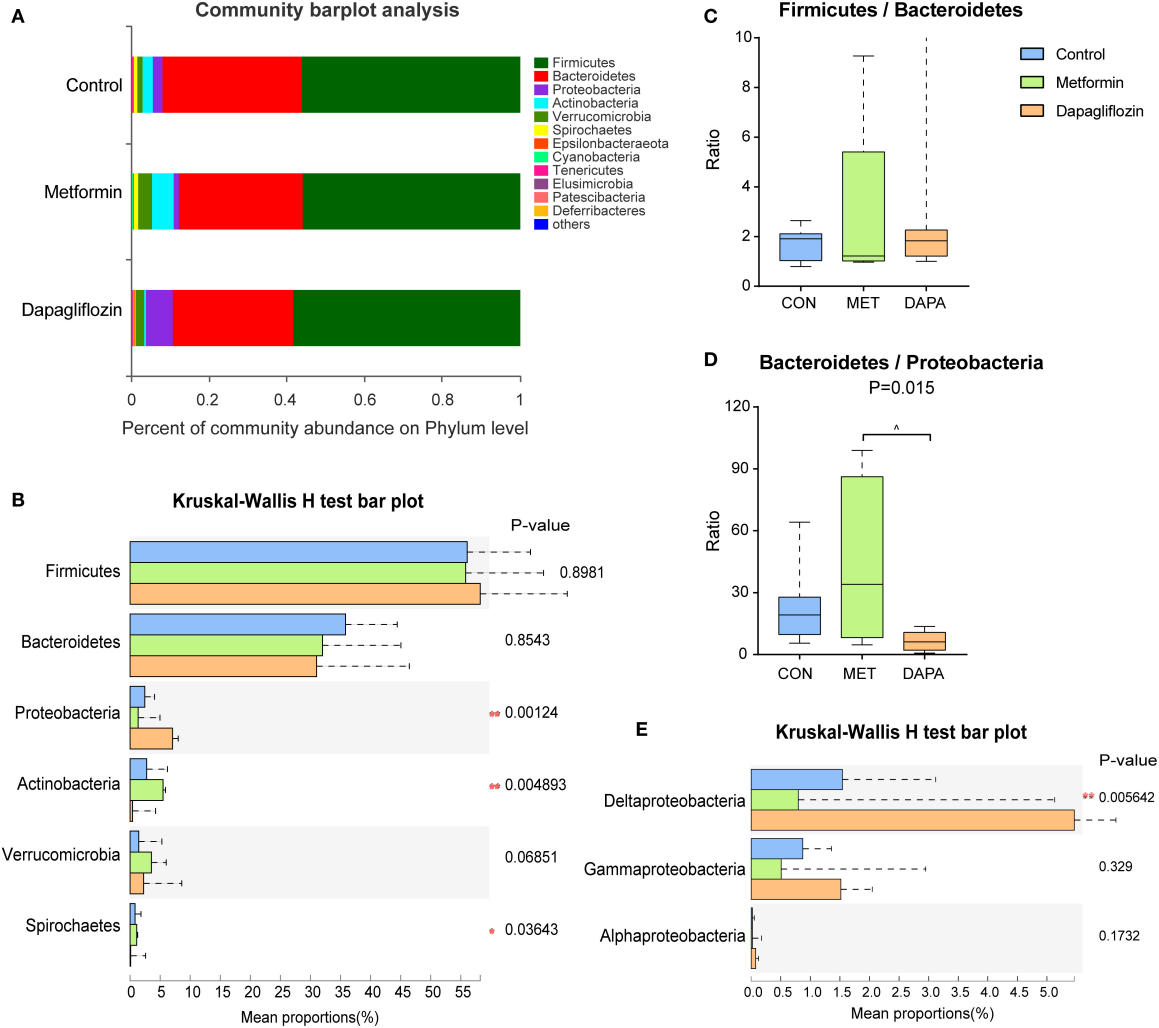
Characterization of core microbial communities. **(A)** Community barplot analysis showing the relative abundance on the phylum level. **(B)** *Firmicutes* to *Bacteroidetes* ratio. **(C)** Relative abundance of the domain phyla *Firmicutes, Bacteroidetes, Proteobacteria, Actinobacteria, Verrucomicrobia*, and *Spirochaetes*. **(D)** Ratio of *Bacteroidetes* to *Proteobacteria*. Kruskal-Wallis rank-sum test was used to assess the ratio of *Firmicutes* to *Bacteroidetes* and the ratio of *Bacteroidetes* to *Proteobacteria*. **(E)** Relative abundance of *Proteobacteria* (delta, gamma, and alpha). *P < 0.05, **P ≤ 0.01, ^metformin versus dapagliflozin *P* < 0.05.

The six major phyla dominated in microbiota were *Firmicutes, Bacteroidetes, Proteobacteria, Actinobacteria, Verrucomicrobia*, and *Spirochates* (Figures 3A,B). The abundance of *Firmicutes* and *Bacteroidetes* did not change significantly among the three groups (Figure 3B) and the ratio of *Firmicutes* to *Bacteroidetes* also exhibited no significant differences (Figure 3C). Type 2 diabetic rats treated with dapagliflozin showed a higher abundance of *Proteobacteria* (*P* < 0.05, Figure 3B), mainly *Proteobacteria* (Delta) but not *Proteobacteria* (Gramma and Alpha) (Figure 3E). The ratio of *Bacteroidetes/Proteobacteria* was decreased in the dapagliflozin group as compared with metformin (Figure 3D). Meanwhile, dapagliflozin lowered the abundance of *Actinobacteria* and *Spriochaetes* compared with metformin treatment (Figure 3B).

**Figure 4:**
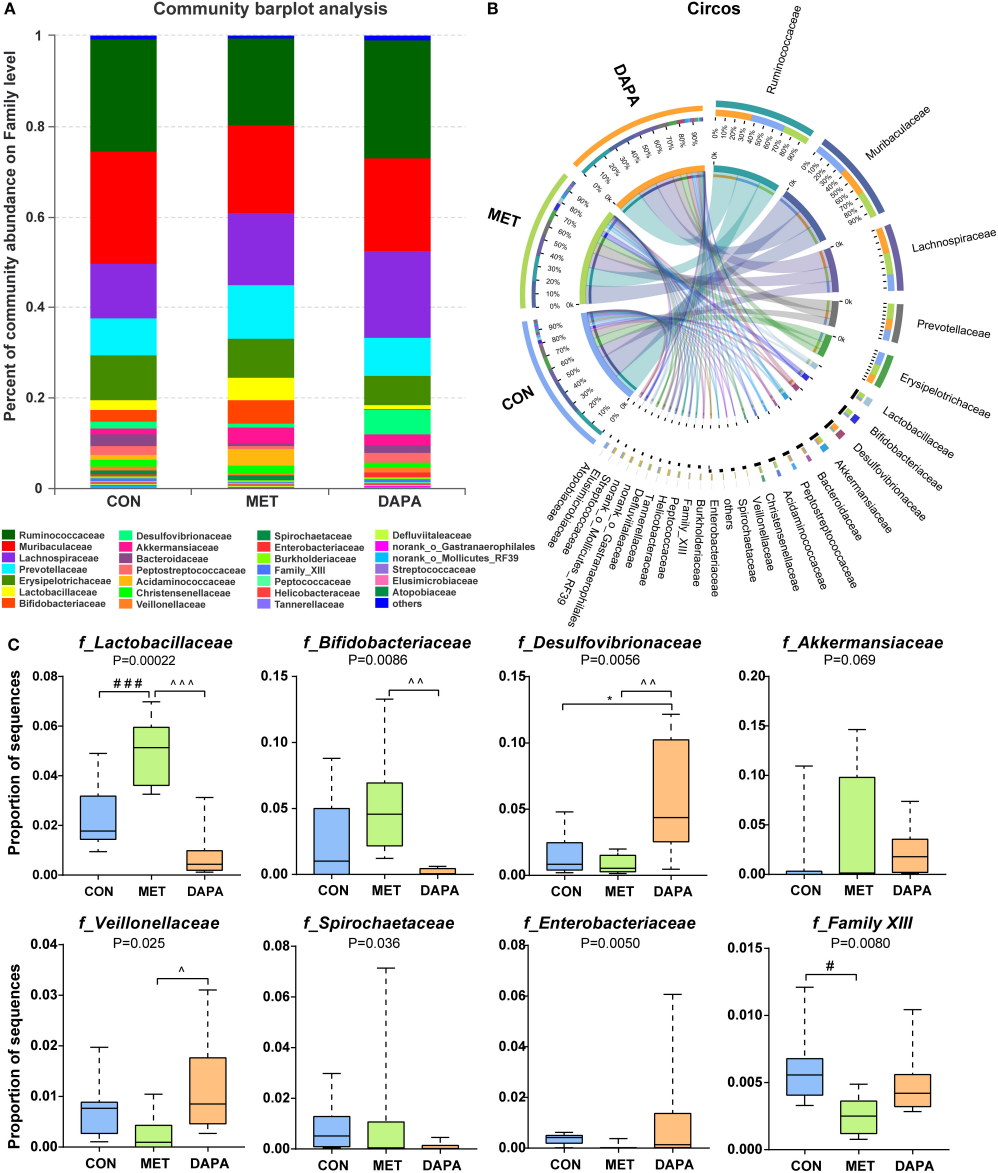
Baterial community abundance on the family level of each group. **(A)** Community barplot demonstrating the relative abundance on the family level. **(B)** Circos plot displaying the relationship between samples and bacteria. **(C)** Relative abundance of core microbial families. * control versus dapagliflozin; # control versus metformin; ^metformin versus dapagliflozin. One special character meant *P* < 0.05, two meant *P* ≤ 0.01 and three meant *P* ≤ 0.001.

**Figure 5:**
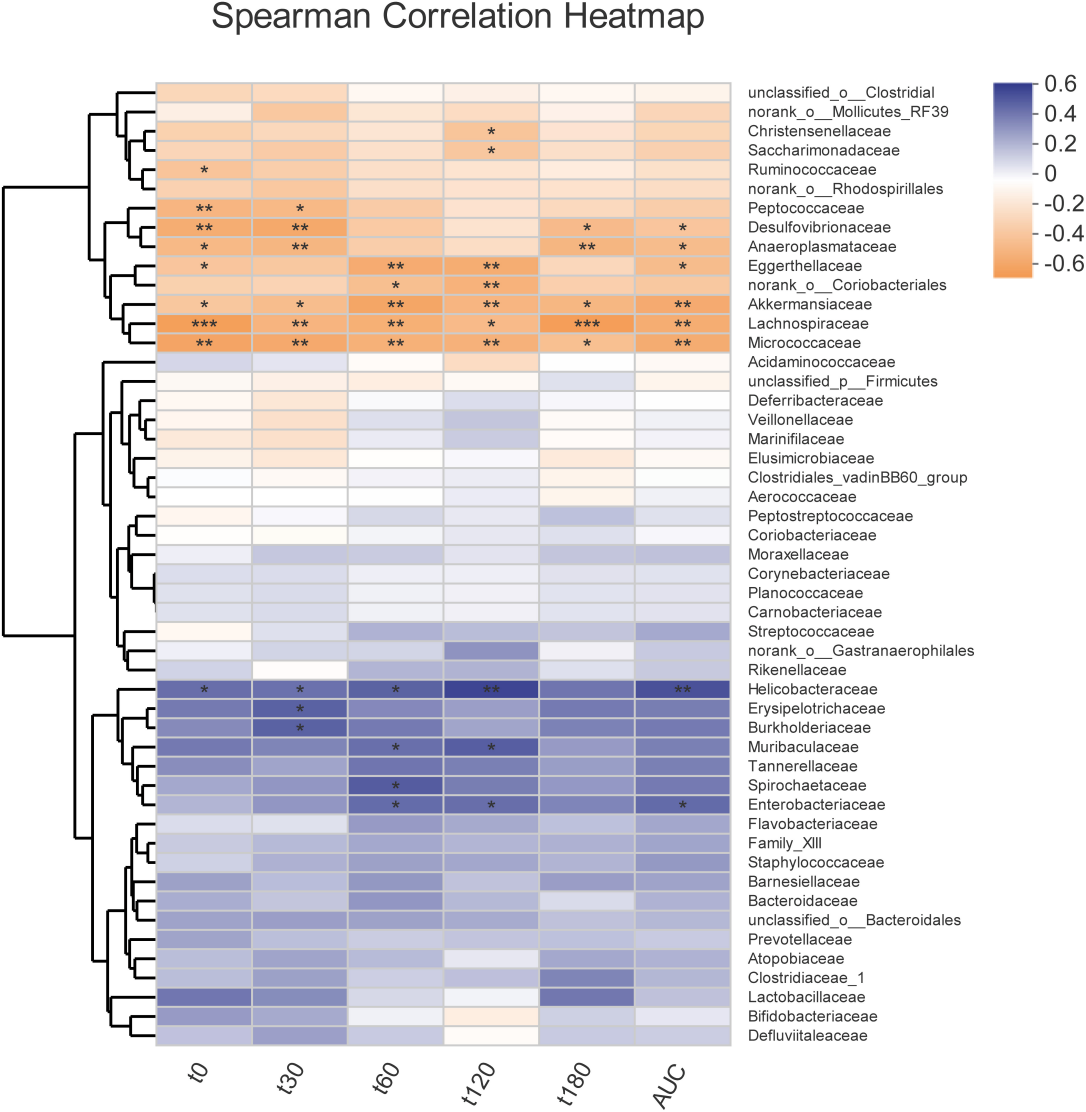
Correlation heatmap of the fecal microbiota with blood glucose indexes. Spearman correlation analysis between the top 50 most abundant bacterial families and blood glucose levels during intragastric glucose tolerance test. *P < 0.05, **P ≤ 0.01, ***P ≤ 0.001.

Overall, community diversity was different among the three groups on the family level (Figure 4, Supplementary Figure 2). The analysis of community relative abundance demonstrated that *Lactobacillaceae* was increased in the metformin group, but decreased in the dapagliflozin group (Figure 4C). Metformin also increased the relative abundance of *Bifidobacteriaceae* and decreased that of *Veillonellaceae* compared with dapagliflozin. *Desulfovibrionaceae* were increased in the dapagliflozin group as compared with the metformin and control group (Figure 4C). Spearman's correlations between the relative abundance of bacterial families and blood glucose were performed. A negative correlation between *Desulfovibrionaceae* and blood glucose was indicated, especially in the fasting blood glucose and 30 min postload (*P* < 0.01) (Figure 5).

**Figure 6:**
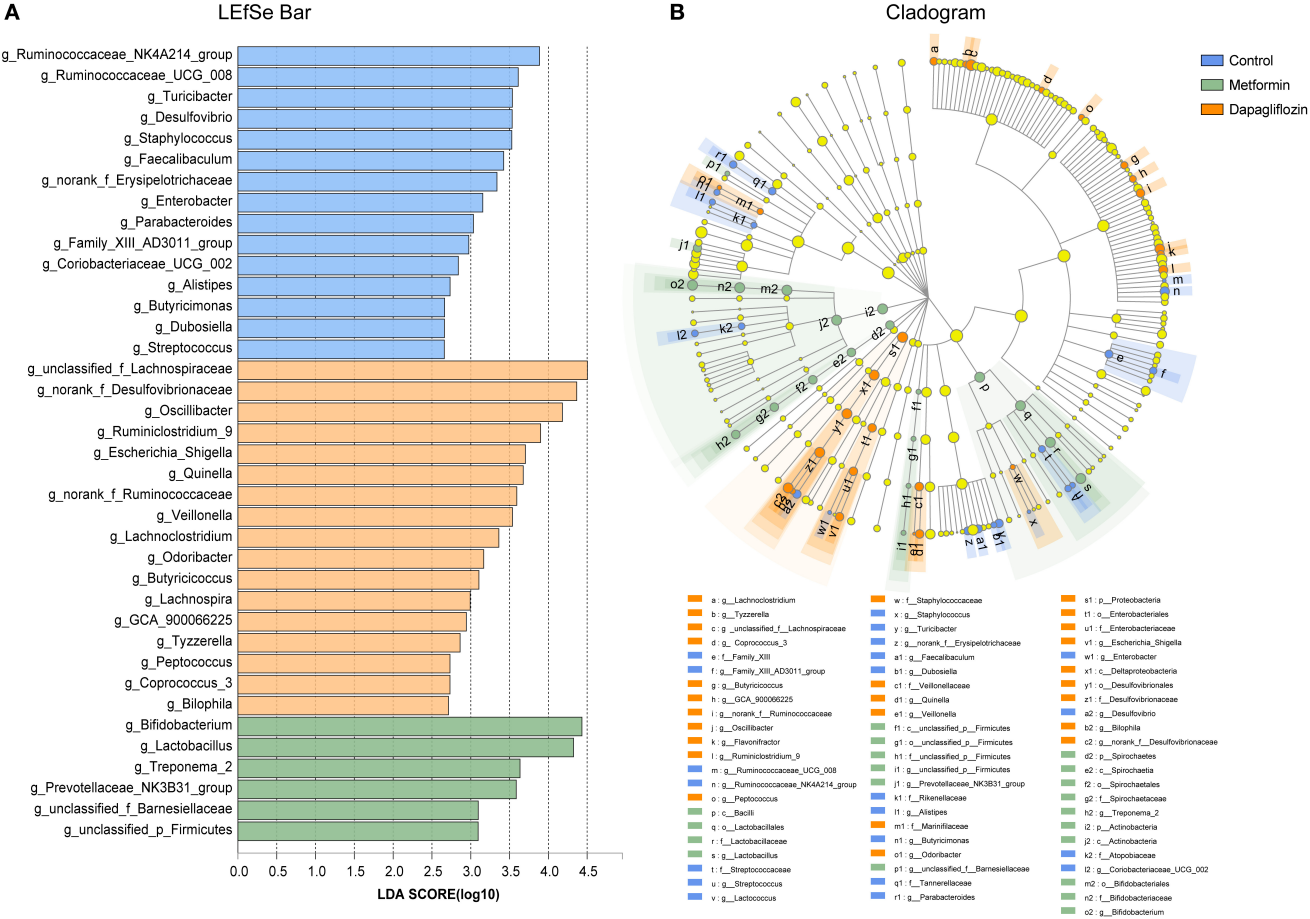
Linear discriminant analysis (LDA) effect size (LEfSe) analysis on genus level among control, metformin, and dapagliflozin group. **(A)** LEfSe barplot on the bacterial genus level. **(B)** Cladogram demonstrating the taxonomic levels with phyla in the innermost and genera in the outermost ring. Only LDA score > 2 are shown. All against all as the multiple comparisons. The prefixes “p” represented as phylum, “c” as class, “o” as order, “f” as family, and “g” as genus.

The linear discriminant analysis (LDA) effect size (LEfSe) revealed that genus, including *Bifidobacterium, Lactobacillus*, and *Treponema 2* were predominant in rats treated with metformin (Figure 6). *Lachnoapiraceae, Desulfovibrionaceae, Oscillibacter, Ruminiclostridium 9* were enriched in the dapagliflozin group (Figure 6).

### 16S rRNA Functional Prediction

By using the PICRUSt and KEGG database on KEGG level 3, glycerolipid metabolism and ion-coupled transporters were enriched in the dapagliflozin group. The Metformin group had more reads which involved purine metabolism and mismatch repair (Supplementary Figure 3A). Concerning the KEGG pathway, glycerolipid metabolism (KO00561) and cytochrome P450 (KO00980) were enriched in dapagliflozin group and three functional categories, purine metabolism (KO00230), nucleotide excision repair (KO03420), and glutathione metabolism (KO00480) (Supplementary Figure 3B), were enriched in the metformin group.

## Discussion

Our study demonstrated that dapagliflozin and metformin monotherapy lowered blood glucose levels to a similar extent. They differentially affected the diversities, and composition of fecal microbiota in type 2 diabetic rats. Of interest, is the result that dapagliflozin had no effects on beneficial bacteria (the abundance of *Lactobacillaceae, Bifidobacteriaceae*, and *Akkermansiaceae* when compared with control) and that it increased the relative abundance of *Proteobacteria* (especially *Desulfovibrionaceae*).

In our study, dapagliflozin did not increase diversity and richness compared with control via alpha diversity analysis, although it has been indicated that dapagliflozin could reduce richness and diversity in mice (13). This result was in line with a recent human study (14). The reason might be the length and dosage difference of the drug intervention (4 weeks vs. 8 weeks; 1 mg/kg/day vs. 60 mg dapagliflozin /kg diet). Beta-diversity analysis and enterotypes were used to further analyze core microbial composition after different treatments.

The concept of “enterotypes” was driven to get an overview of the robust clustering of samples species variation (18). Dapagliflozin and metformin had differential effects on the composition of fecal microbiota enterotypes on the family level. The Dapagliflozin group mainly displayed with *Ruminococcaceae* and the metformin group presented *Ruminococcaceae* and *Muribaculaceae* (18). *Ruminococcaceae* was regarded as certain SCFA-producing bacteria (19). A *Muribaculaceae-*history named the S24-7 belongs to the phylum *Bacteroidate*s (20) and it has been suggested that *Muribaculaceae* abundance is an important predictor of SCFAs levels (21).

*Firmicutes* and *Bacteroidetes* are the predominant phyla in gut microbiota, and account for around 90% of the known phylogenetic categories (22). The ratio of *Firmicutes* to *Bacteroidetes* was positively related to fecal SCFAs (23). A Danish study found that the ratio of *Firmicutes* to *Bacteroidetes* in patients with type 2 diabetes was lower than those in non-diabetic subjects (24, 25). It has been verified that the ratio of *Firmicutes* to *Bacteroidetes* was also negatively correlated to fasting blood glucose levels and inflammation status (26). T2DM, induced by HFD and STZ, presented higher contents of pro-inflammatory cytokines and insulin resistance, accompanied by a larger proportion of *Firmicutes* and a relatively lower *Bacteroidetes* in the fecal microbiota (27). We did not show any differences in the ratio of *Firmicutes* to *Bacteroidetes*, although Lee et al. (13) reported that the ratio of *Firmicutes* to *Bacteroidetes* was decreased in diabetic mice after dosage with dapagliflozin. Our findings were consistent with a short time intervention study, which showed that canagliflozin, did not influence the abundance of the *Firmicutes* to *Bacteroidetes* ratio (12).

*Proteobacteria* are enriched in the fecal microbiota of patients with obesity and T2DM (28). *Desulfovibrionaceae* belongs to the phylum *Proteobacteria (Delta)* (29). We demonstrated that dapagliflozin increased the relative abundance of *Proteobacteria (especially Delta)* and *Desulfovibrionaceae* compared with the metformin and control group. This study firstly presented that dapagliflozin increased *Desulfovibrionaceae*, but revealed that, on the contrary, metformin decreased *Desulfovibrionaceae* which was reported in metabolically dysfunctional mice (30).

*Actinobacteria* is a group of Gram-positive bacteria (31). *Bifidobacteriaceae* belong to *Actinobacteria*, and this was considered to improve metabolic endotoxemia and glucose intolerance (32). The abundance of *Bifidobacteriaceae* was lower in patients with diabetes (32). It has been reported that canagliflozin increased the abundance of *Bifidobacterium* in mice with chronic kidney disease (12). Dapagliflozin is a much weaker SGLT1 inhibitor as compared with canagliflozin (33), which suppresses SGLT1 by 40–60% (34). Notably, we showed that dapagliflozin and metformin exerted a complementary effect in the relative abundance of *Actinobacteria* and *Bifidobacteriaceae*. They were decreased in dapagliflozin while increased in the metformin group. This implied that the combination therapy of dapagliflozin and metformin might be of clinical significance.

Recently, it was revealed that dapagliflozin sustained beta cell mass (35). Our study found that dapagliflozin lowered similar blood glucose levels to metformin and further improved insulin resistance. However, the relationship between beta cell function and fecal microbiota remains uncertain. Whether fecal microbiota improves pancreatic beta cell growth by generating SCFAs needs to be further clarified (36).

This study has several limitations. Firstly, 16S rRNR gene sequencing rather than metagenomics sequencing was used, which limits species level and function analysis on data interpretation. Secondly, we employed HFD and STZ induced diabetic model, which might be a lack of genetic influence. It might be different in the distribution of the fecal microbiota from other diabetic models such as Zucker diabetic fatty rats in our previous study (16). Thirdly, the model of HFD and low-dose STZ to induce a diabetic model might have some deficiencies, as STZ can destroy the beta cells thereby increasing hyperglycemia. However, this is not just the same case in human T2D since the insulin secretion is unchanged or increased. As diabetes progressed, the function of beta cells in some diabetic patients also declined, but there are differences between animal studies and patients with type 2 diabetes. Therefore, the results of our study may need to be verified by diabetic patients. Finally, this study was only a preliminary observation, and the mechanism needs to be further explored. In future studies, we would like to detect the roles of the *Desulfovibrionaceae* on the pathophysiology and mechanism of diabetes and obesity.

In conclusion, our study demonstrated preliminary results that dapagliflozin had no effects on the abundance of beneficial bacteria. Of interest, is the observation that dapagliflozin enriched *Proteobacteria* (especially *Desulfovibrionaceae*), which were associated with obesity and diabetes. Combination dapagliflozin with metformin might have complementary effects on fecal microbiota in the treatment of diabetes.

## Data Availability Statement

The datasets presented in this study can be found in online repositories. The names of the repository/repositories and accession number(s) can be found below: NCBI BioProject [accession: PRJNA649663].

## Ethics Statement

The animal study was reviewed and approved by Institutional Animal Care and Use Committee of SLAC.

## Author Contributions

MY conducted the animal experiments. F-HS wrote the manuscript, modifications, and submission. WenL wrote the methods and results. M-CZ analyzed the data and prepared the figures. R-LF and CQ performed the fecal DNA extraction. WeiL contributed to the study design and review the manuscript. JM was the guarantor of this study who has full access to all the data to guarantee the accuracy and integrity of the data. All authors contributed to the article and approved the submitted version.

## Conflict of Interest

The authors declare that the research was conducted in the absence of any commercial or financial relationships that could be construed as a potential conflict of interest.
