# Supplementary material for: Dapagliflozin Modulates the Fecal Microbiota in a Type 2 Diabetic Rat Model
Source: Front Endocrinol (Lausanne). 2020 Nov 17;11:635. doi: 10.3389/fendo.2020.00635 (PMC7707060; doi:10.3389/fendo.2020.00635)

Figure.S1. The effects of dapagliflozin and metformin on blood glucose levels (A and B) and HOMA-IR (C) during the intragastric glucose tolerance (IGGTT) after 4 weeks treatments.

Data were expressed as mean ± SEM and assessed by one-way analysis of variance (ANOVA). The statistical significance was presented by different characters as * control versus dapagliflozin; # control versus metformin; ^ metformin versus dapagliflozin. One special character meant P < 0.05, two meant P < 0.01 and three meant P ≤ 0.001.

Figure S2 Community heatmap showing abundant microbes on family level. One column represents one samples. C: control; M: metformin; D: dapagliflozin

Figure S3 Functional profiling of KEGG in metagenomic predictions by using Phylogenetic Investigation of Communities by Reconstruction of Unobserved States (PICRUSt) and LDA effect size (LEfSe) analysis. LEfSe barplot showing different abundance of (A) KEGG level 3 and (B) KEGG pathway. Only log linear discriminant analysis (LDA) >2 was shown.

Figure S1


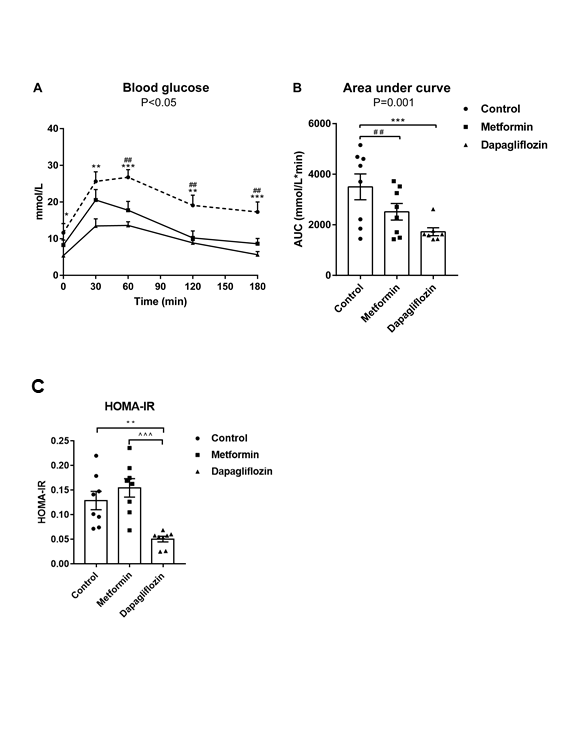


Figure S2


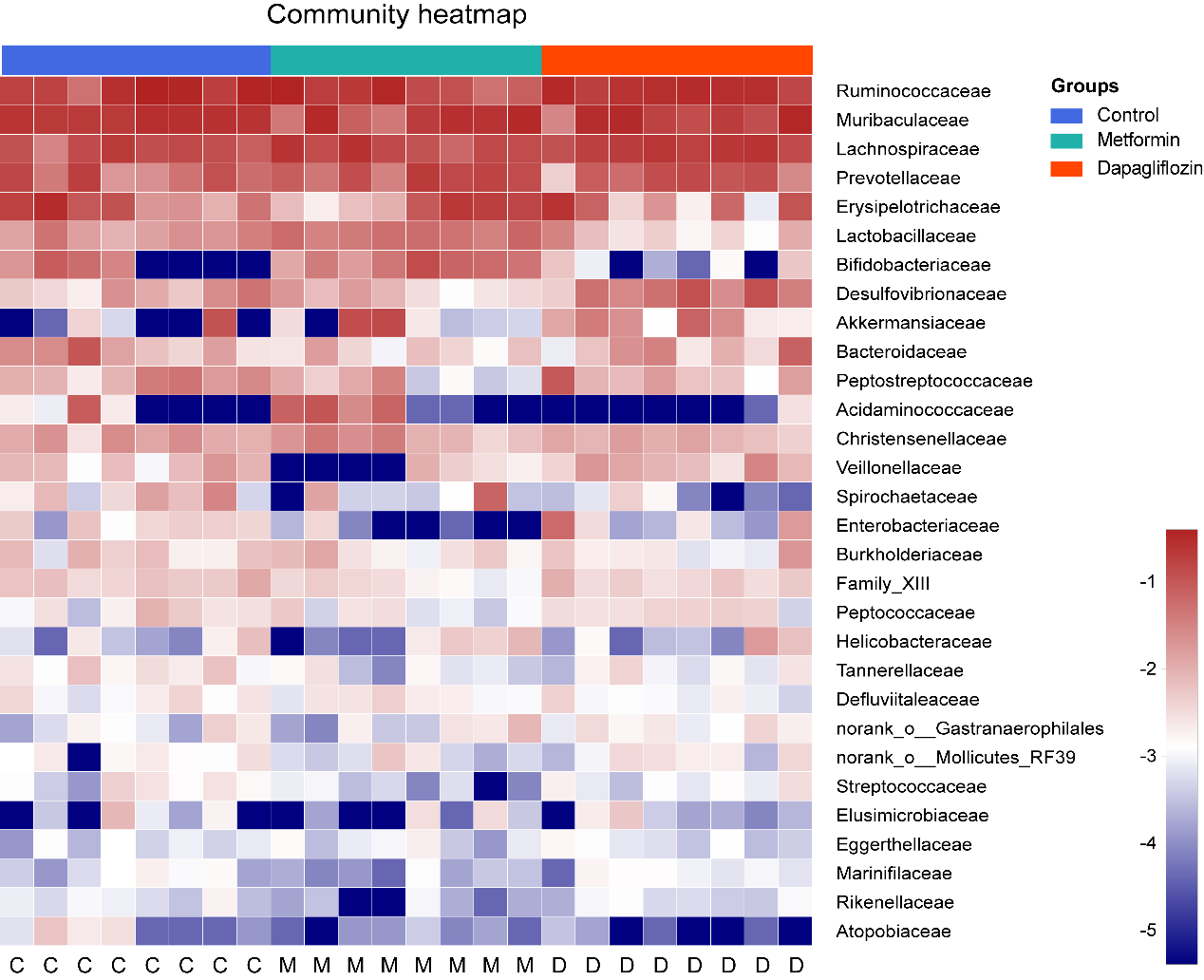


Figure S3


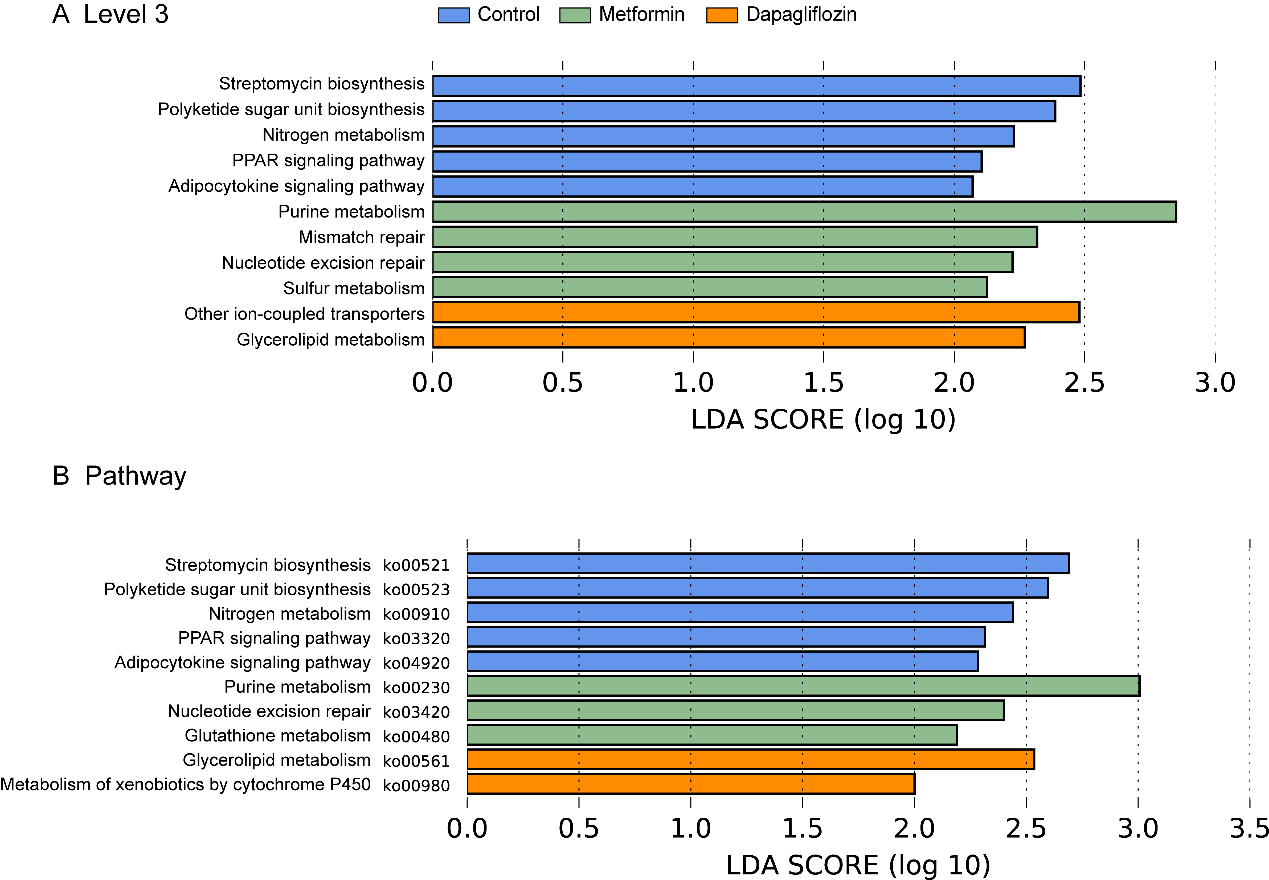

Supplement: Supplementary file 1 [file Data_Sheet_1.docx]
